# Personality Traits in Patients with Depression: Association with Symptoms of Depression and Anxiety

**DOI:** 10.1192/j.eurpsy.2024.543

**Published:** 2024-08-27

**Authors:** S. Galnaitytė, V. Adomaitienė

**Affiliations:** ^1^Faculty of Medicine, Vilnius University, Vilnius; ^2^Clinic of Psychiatry, Lithuanian University of Health Sciences, Kaunas, Lithuania

## Abstract

**Introduction:**

The symptoms of depression and anxiety, which are frequently comorbid, may be significantly impacted by the individual’s personality, even considering the complex etiology of depression. Several studies have shown that while certain personality traits may act as protective factors, others may increase vulnerability to depression and anxiety. Understanding these relationships may be important since personality traits have gained attention as potential determinants of symptom severity and treatment outcomes.

**Objectives:**

To identify and evaluate the association of personality traits with symptoms of depression and anxiety in patients with depression.

**Methods:**

The study involved 80 inpatients (≥ 18 years), hospitalised in University psychiatry department with depression diagnosis based on the ICD-10-AM classification. Subjects were asked to fill the Overall Anxiety Severity and Impairment Scale (“OASIS”), the Big Five Personality Dimensions scale and the Patient Health Questionnaire-9 (PHQ-9). Data analysis included descriptive data, Shapiro-Wilk test, Spearman correlation, Kruskal–Wallis test and Chi-Square test, with a significance threshold of p<0.05.

**Results:**

Severe (26.3%) and very severe (41.3%) depressive symptoms were the most prevalent. Extraversion was associated with minimal (p=0.002), conscientiousness with mild (p<0.001), neuroticism with very severe depressive symptoms (p=0.003). The majority of depressed patients had severe (33,75 %) or very severe (32,5 %) anxiety symptoms. Anxiety symptoms were associated with more severe depressive symptoms (r=0.704, p<0.001). The association of conscientiousness and moderate anxiety symptoms was found (p=0.004). In the presence of expressed neuroticism, most of the respondents showed very severe anxiety symptoms, in the absence of neuroticism – moderate anxiety symptoms (p<0.001).

**Conclusions:**

The results showed that personality traits were associated with severity of depression and anxiety symptoms in psychiatry inpatient with depression. Therefore, recognition of predominant personality traits in patients with depression may be helpful in selecting treatment and predicting treatment outcomes.

**Disclosure of Interest:**

None Declared

